# Factors Associated With Mechanical Ventilation in Patients Hospitalized With Hepatic Encephalopathy: A Nationwide Inpatient Sample Analysis

**DOI:** 10.7759/cureus.108332

**Published:** 2026-05-05

**Authors:** Idan Grossmann, Harshavardhan Sanekommu, Muhammad Faiq Akram, Rimsha Riaz, Urvah Tauseef, Natasha Campbell, Bhaveshkumar Garsondiya, Shuvendu Sen, Mohammad A Hossain, Lee Peng

**Affiliations:** 1 Internal Medicine, Hackensack Meridian Health Jersey Shore University Medical Center, Neptune, USA; 2 Gastroenterology and Hepatology, Hackensack Meridian Health Jersey Shore University Medical Center, Neptune, USA; 3 Internal Medicine, Allama Iqbal Medical College, Lahore, PAK; 4 Internal Medicine, Faisalabad Medical University, Faisalabad, PAK

**Keywords:** clinical predictors, decompensated liver cirrhosis, hepatic encephalopathy, liver cirrhosis, mortality, nationwide inpatient sample analysis

## Abstract

Background

Hepatic encephalopathy (HE) is a debilitating and potentially fatal complication of advanced liver disease. While most patients with HE improve with medical therapy, a significant subset requires mechanical ventilation, an escalation of care associated with higher mortality, prolonged hospitalization, and greater financial burden. Despite this clinical and economic burden, factors associated with mechanical ventilation and the outcomes of intubated HE patients remain poorly defined at a national level. We aimed to characterize both the risk factors for mechanical ventilation and the associated clinical outcomes among hospitalized patients with HE using a large, representative cohort.

Methods

We performed a retrospective cohort study using the National Inpatient Sample (NIS) from 2016 to 2020. Adult patients (≥18 years) hospitalized with HE were included and identified using International Classification of Diseases, 10th Revision (ICD-10) codes for cirrhosis with HE. Elective admissions and transfers from acute-care hospitals were excluded. Baseline characteristics and clinical outcomes were compared between patients requiring mechanical ventilation and those not intubated. Multivariable logistic regression was used to identify factors associated with mechanical ventilation.

Results

From 2016 to 2020, 572,600 HE hospitalizations were identified, with 9.1% (52,295) requiring mechanical ventilation. Ventilated patients were younger (57.9 vs. 60.3 years, p<0.001), more often male (61% vs. 58%) and Black (11% vs. 8.6%), and had higher rates of sepsis (50% vs. 12%), acute kidney injury (AKI) (64% vs. 35%), and gastrointestinal (GI) bleeding (28% vs. 16%) (p<0.001 for all). Mechanical ventilation was associated with higher mortality (43% vs. 4.7%; adjusted OR, 8.32; 95% CI, 7.84-8.83), longer length of stay (11.5 vs. 6.0 days; adjusted RR, 1.43; 95% CI, 1.40-1.47), and increased costs ($48,511 vs. $15,731; adjusted RR, 2.23; 95% CI, 2.18-2.28), all p<0.001. On multivariable analysis, the strongest factors associated with mechanical ventilation included sepsis (OR, 5.42; 95% CI, 5.17-5.67), AKI (OR, 2.35; 95% CI, 2.24-2.46), and GI bleeding (OR, 1.84; 95% CI, 1.75-1.94).

Conclusion

Nearly 1 in 10 hospitalized patients with HE required mechanical ventilation, which was associated with substantially higher in-hospital mortality and increased hospitalization costs. Sepsis, renal failure, and GI bleeding were the strongest factors associated with intubation. These findings suggest that mechanical ventilation likely reflects underlying disease severity rather than serving as an independent driver of mortality. These clinical features are associated with mechanical ventilation and may aid risk stratification in hospitalized patients with HE.

## Introduction

Liver cirrhosis represents the end stage of chronic liver disease and is characterized by diffuse hepatic fibrosis resulting from persistent liver injury and inflammation due to multiple etiologies, including chronic viral hepatitis, metabolic dysfunction-associated steatotic liver disease, and alcohol-associated liver disease [1]. Hepatic encephalopathy (HE) is a major neuropsychiatric complication of cirrhosis resulting from impaired hepatic detoxification and portosystemic shunting, leading to the accumulation of neurotoxins, such as ammonia, that disrupt cerebral function [2]. Clinically, HE encompasses a spectrum of neurological disturbances ranging from subtle cognitive impairment to profound alterations in consciousness and coma. The development of HE is associated with substantial morbidity, recurrent hospitalizations, impaired quality of life, and increased mortality among patients with advanced liver disease [3].

The burden of HE-related hospitalizations has risen steadily over the past decade, making it a major contributor to healthcare utilization and costs among patients with cirrhosis [4]. In these critically ill individuals, progressive encephalopathy may result in loss of airway protection, respiratory compromise, and the need for invasive mechanical ventilation. The requirement for mechanical ventilation represents a significant escalation of care and is frequently associated with worse clinical outcomes, prolonged hospitalization, and increased healthcare expenditures [5]. Episodes of HE are commonly precipitated by acute systemic insults, including infections, gastrointestinal bleeding, and renal dysfunction. These complications not only trigger or exacerbate encephalopathy but may also contribute to systemic instability and respiratory failure requiring ventilatory support [6].

Despite the clinical significance of mechanical ventilation in this population, no prior nationwide study has specifically evaluated factors associated with mechanical ventilation among patients hospitalized with HE, and the subsequent impact on clinical outcomes remains incompletely defined.

Prior studies examining outcomes in cirrhosis and HE have largely focused on overall mortality and hospitalization trends, with limited attention to the determinants and consequences of mechanical ventilation in this population [7]. Furthermore, existing data are frequently derived from single-center cohorts or relatively small study populations, limiting their generalizability [8]. Understanding factors associated with mechanical ventilation in this population may help improve risk stratification, guide early clinical decision-making, and optimize healthcare resource utilization in a high-cost inpatient setting. Using the National Inpatient Sample (NIS), this study aimed to identify factors associated with mechanical ventilation among hospitalized patients with HE and to evaluate associated in-hospital outcomes, including mortality, length of stay, and hospitalization costs [9].

## Materials and methods

Data availability and data source

We retrospectively evaluated data from 2016 to 2020 extracted from the NIS, a component of the Healthcare Cost and Utilization Project (HCUP), facilitated by the Agency for Healthcare Research and Quality. The NIS is a publicly available all-payer inpatient healthcare database, including Medicaid, Medicare, private insurance, and self-pay, that approximates a 20% stratified sample of all annual hospitalizations from non-federal hospitals in the US across 49 states, covering around 98% of the US population.

The database includes de-identified patient information, such as demographics, hospital characteristics, length of stay (LOS), mortality, and hospital charges, including up to 40 diagnoses and 25 procedure codes from International Classification of Diseases, 10th Revision (ICD-10). The NIS represents hospitalization-level encounters rather than patient-level data; therefore, multiple admissions for the same patient could not be tracked. Approval from the Institutional Review Board (IRB) was not required for this retrospective study given its use of anonymous, publicly available data.

Study population

Adult patients aged ≥18 years hospitalized with HE were identified using ICD-10 codes for cirrhosis and HE. HE and cirrhosis were identified using ICD-10 diagnosis codes in any position, primary or secondary, and patients were included if at least one code for HE and one code for cirrhosis were present during the same hospitalization. Patients were identified using primary and secondary ICD-10 diagnosis codes, with at least one relevant code required for inclusion. Younger patients aged <18 years, elective admissions, and transfers from other acute-care hospitals were excluded. The patients were stratified into two groups: those who required intubation and mechanical ventilation and those who did not. The full list of ICD-10 codes used is provided in Table 1.

**Table 1:** ICD-10 codes used to define study variables and comorbid conditions. ICD-10: International Classification of Diseases, 10th Revision.

Variable	ICD-10 codes
Cirrhosis	K71.7, K74.3, K74.4, K74.5, K70.30, K70.31, K74.60, K74.69
Hepatic encephalopathy	K72.91, K72.01, K72.11, K72.90
Intubation/mechanical ventilation	5A1935Z, 5A1945Z, 5A1955Z
Acute kidney injury (AKI)	N17
Diabetes mellitus	E10.0, E10.1, E10.6, E10.8, E10.9, E11.0, E11.1, E11.6, E11.8, E11.9, E12.0, E12.1, E12.6, E12.8, E12.9, E13.0, E13.1, E13.6, E13.8, E13.9, E14.0, E14.1, E14.6, E14.8, E14.9
Diabetes mellitus with complications	E10.2-E10.5, E10.7, E11.2-E11.5, E11.7, E12.2-E12.5, E12.7, E13.2-E13.5, E13.7, E14.2-E14.5, E14.7
Hyperlipidemia	E78
Sepsis	A02.1, A22.7, A26.7, A32.7, A40.0, A40.1, A40.3, A40.8, A40.9, A41.01, A41.02, A41.1, A41.2, A41.3, A41.4, A41.50, A41.51, A41.52, A41.53, A41.59, A41.81, A41.89, A41.9, A42.7, A54.86, B37.7, R65.20, R65.21
Gastrointestinal bleeding	K92.2, K92.0, K92.1, I85.0, I85.00, I85.01, I98.20, I98.3, K22.10, K22.12, K22.14, K22.16, K25.0, K25.2, K25.4, K25.6, K26.0, K26.2, K26.4, K26.6, K27.0, K27.2, K27.4, K27.6, K28.0, K28.2, K28.4, K28.6, K29.0, K63.80, K31.80, K55.20, K62.5
Ascites	R18.8
Alcohol abuse, uncomplicated	F10.10

Outcomes

The primary outcomes evaluated were in-hospital mortality, LOS, and total hospitalization charges. Secondary outcomes included baseline characteristics, such as age, gender, insurance type, hospital bed size, and hospital location; Elixhauser comorbidity score; diagnosis of alcohol use disorder (AUD); acute kidney injury (AKI) on admission; sepsis; diabetes mellitus; ascites; GI bleeding; and diagnosis of hyperlipidemia. The Elixhauser comorbidity index was derived using the HCUP Elixhauser Comorbidity Software based on ICD-10 diagnosis codes.

Statistical analysis

All analyses were conducted using data weighted according to the appropriate NIS discharge weights to obtain nationwide estimates. Multivariable logistic regression was used to identify factors associated with mechanical ventilation. Covariates included in the multivariable logistic regression model were selected a priori based on clinical relevance and prior literature and included age, sex, race, insurance status, hospital characteristics, Elixhauser comorbidity score, and relevant comorbid conditions. No stepwise selection procedures were used. Multicollinearity was assessed using variance inflation factors, and no significant collinearity was identified. Statistical analyses were performed using Stata version 17 (StataCorp LLC, TX, USA).

The NIS uses a complex survey design that incorporates stratification, clustering, and weighting. All analyses were performed using survey-weighted procedures to account for hospital-level clustering and to generate nationally representative estimates.

Due to limitations of the dataset, markers of liver disease severity, including the Model for End-Stage Liver Disease (MELD) score and Child-Pugh classification, were not available for adjustment in the regression analysis.

Additionally, because of the cross-sectional nature of the database, the temporal relationship between complications such as sepsis, AKI, and mechanical ventilation cannot be determined; therefore, reverse causality cannot be excluded.

Sensitivity analyses were not performed due to constraints of the dataset structure and the predefined exposure and outcome definitions within the NIS.

Missing data were assessed for all covariates included in the analysis. The overall proportion of missingness across variables was <5%. Specifically, missingness was <1% for age, sex, race, insurance status, hospital characteristics, and Elixhauser comorbidity score. Clinical variables, including sepsis, AKI, GI bleeding, diabetes mellitus, ascites, and hyperlipidemia, had missingness ranging from 0.2% to 4.1%. Given the low proportion of missing data across all variables, a complete-case analysis approach was used. Missingness was assumed to be random and unlikely to introduce meaningful bias into the regression estimates.

## Results

Patients

**Figure 1:**
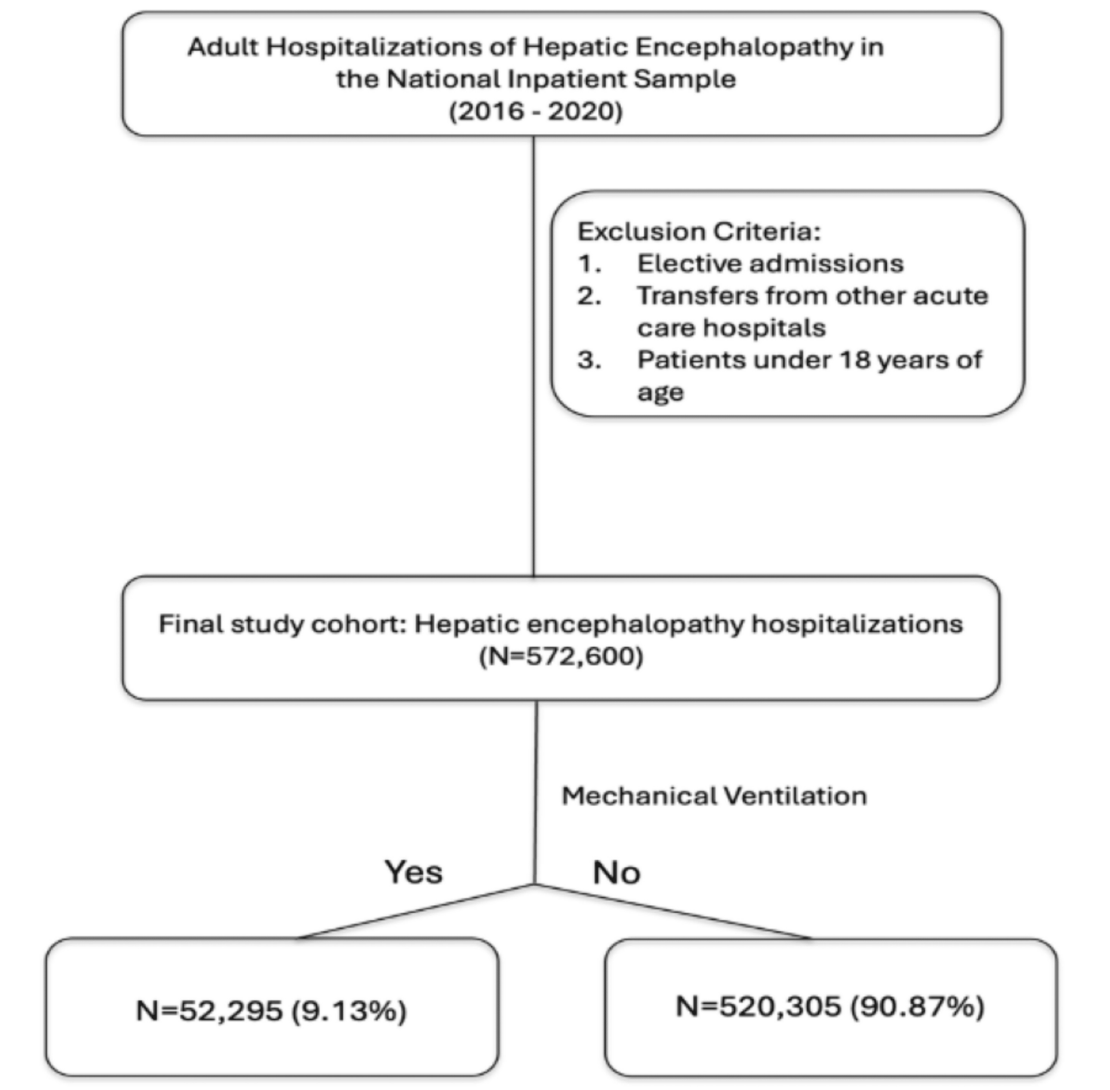
Mechanical ventilation among hospitalized patients with hepatic encephalopathy.

A total of 572,600 hospitalizations with HE were identified in the NIS between 2016 and 2020. Among these, 52,295 patients (9.1%) required mechanical ventilation, whereas 520,305 (90.9%) were managed without intubation (Figure 1). Compared with non-intubated patients, those who underwent mechanical ventilation were younger (mean age, 57.9 vs. 60.3 years) and more often male (61% vs. 58%). Black patients comprised a higher proportion of the ventilated group (11% vs. 8.6%), whereas the relative proportions of Hispanic, Asian or Pacific Islander, and Native American patients were broadly similar between groups. Patients requiring mechanical ventilation had a higher comorbidity burden, with a greater mean Elixhauser comorbidity score than non-intubated patients (6.2 vs. 5.5).

Insurance coverage and hospital characteristics also differed by intubation status, with intubated patients less likely to have Medicare (39% vs. 48%) and more likely to have Medicaid (31% vs. 25%), private insurance (21% vs. 19%), or other insurance (9.3% vs. 7.5%). They were also more often admitted to large hospitals (54% vs. 52%) and urban teaching centers (75% vs. 71%) and less often admitted to rural hospitals (4.1% vs. 7.3%) (Table 2). The extent of missing data across included variables was low and did not result in substantial exclusion of hospitalizations from the final analysis. No individual variable exceeded 4.1% missingness.

**Table 2:** Baseline characteristics of hospitalizations for hepatic encephalopathy, stratified by mechanical ventilation status. Baseline characteristics of hospitalizations for hepatic encephalopathy, stratified by mechanical ventilation status. Continuous variables are presented as mean (SE), and categorical variables are presented as n (%). P-values indicate differences between intubated and non-intubated hospitalizations.

Characteristic	Overall, N = 572,600	Not intubated, N = 520,305	Intubated, N = 52,295	p-value
Age, mean (SE)	60.1 (0.04)	60.3 (0.05)	57.9 (0.11)	<0.001
Sex (%)				<0.001
Male	331,190 (58%)	299,235 (58%)	31,955 (61%)	
Female	241,345 (42%)	221,015 (42%)	20,330 (39%)	
Race (%)				<0.001
White	365,165 (65%)	333,530 (65%)	31,635 (62%)	
Black	49,510 (8.8%)	43,860 (8.6%)	5,650 (11%)	
Hispanic	108,465 (19%)	98,485 (19%)	9,980 (20%)	
Asian or Pacific Islander	11,105 (2.0%)	9,960 (2.0%)	1,145 (2.2%)	
Native American	9,845 (1.8%)	8,985 (1.8%)	860 (1.7%)	
Other	16,310 (2.9%)	14,560 (2.9%)	1,750 (3.4%)	
Insurance (%)				<0.001
Medicare	271,530 (47%)	251,105 (48%)	20,425 (39%)	
Medicaid	146,735 (26%)	130,770 (25%)	15,965 (31%)	
Private	109,970 (19%)	99,000 (19%)	10,970 (21%)	
Other	43,680 (7.6%)	38,840 (7.5%)	4,840 (9.3%)	
Unknown	685 (0.1%)	590 (0.1%)	95 (0.2%)	
Elixhauser comorbidity score, mean (SE)	5.6 (0.01)	5.5 (0.01)	6.2 (0.02)	<0.001
Hospital bed size (%)				<0.001
Small	106,730 (19%)	98,070 (19%)	8,660 (17%)	
Medium	166,140 (29%)	150,655 (29%)	15,485 (30%)	
Large	299,730 (52%)	271,580 (52%)	28,150 (54%)	
Hospital location/teaching status (%)				<0.001
Urban teaching	409,390 (71%)	370,375 (71%)	39,015 (75%)	
Rural	39,900 (7.0%)	37,735 (7.3%)	2,165 (4.1%)	
Urban nonteaching	123,310 (22%)	112,195 (22%)	11,115 (21%)	

Primary outcomes

Mechanical ventilation was associated with substantially worse in-hospital outcomes. Crude in-hospital mortality was markedly higher among intubated patients than among those not intubated (43% vs. 4.7%, p<0.001). After multivariable adjustment, mechanical ventilation remained strongly associated with in-hospital mortality (adjusted odds ratio [aOR], 8.32; 95% CI, 7.84-8.83; p<0.001).

Resource utilization followed a similar trend in ventilated patients. Mean length of stay was almost doubled (11.5 vs. 6.0 days, p<0.001), corresponding to an adjusted rate ratio (aRR) of 1.43 (95% CI, 1.40-1.47; p<0.001). Mean hospitalization costs were more than threefold higher in the intubated group than in the non-intubated group (48,511 vs. 15,731 US dollars, p<0.001), with an adjusted cost aRR of 2.23 (95% CI, 2.18-2.28; p<0.001) (Table 3).

**Table 3:** Primary outcomes among hospitalizations for hepatic encephalopathy, stratified by intubation status. Continuous variables were compared using a t-test or Kruskal-Wallis test, as appropriate, and categorical variables were compared using the Rao-Scott adjusted chi-square test. Corresponding test statistics, t or χ², are reported alongside p-values. Relative ratios for length of stay (LOS) were estimated using a Poisson regression model, and hospital costs were analyzed using a generalized linear model with gamma distribution and log link. Costs were adjusted for inflation using the Consumer Price Index (CPI) for medical care services in the United States, with 2020 as the reference year. SE: Standard error; LOS: Length of stay; CPI: Consumer Price Index.

Outcome	Not intubated, N = 520,305	Intubated, N = 52,295	p-value*	Adjusted OR/RR	95% CI	p-value
In-hospital mortality, n (%)	24,300 (4.7%)	22,360 (43%)	<0.001	8.32	7.84-8.83	<0.001
LOS, mean (SE)	6.0 (0.02)	11.5 (0.13)	<0.001	1.43	1.40-1.47	<0.001
Hospital cost, mean (SE)	15,731 (118)	48,511 (663)	<0.001	2.23	2.18-2.28	<0.001

Factors associated with mechanical ventilation

In multivariable analysis, we observed modest demographic and systems-level associations with mechanical ventilation. Increasing age was associated with slightly lower odds of intubation (aOR, 0.99 per year; 95% CI, 0.98-0.99), and female sex was associated with lower odds than male sex (aOR, 0.92; 95% CI, 0.88-0.96; both p<0.001). Relative to White patients, Black patients (aOR, 1.17; 95% CI, 1.08-1.26), Asian or Pacific Islander patients (aOR, 1.20; 95% CI, 1.02-1.40), and patients of other races (aOR, 1.23; 95% CI, 1.07-1.40) had higher odds of mechanical ventilation, whereas Native American race was not significantly associated. Insurance and hospital factors showed similarly modest effects: compared with Medicare, Medicaid (aOR, 1.15), private insurance (aOR, 1.13), and other insurance (aOR, 1.23) were associated with higher odds of intubation; medium- and large-bed-size hospitals had higher odds than small hospitals (aOR, 1.14 and 1.18, respectively), while rural hospitals had lower odds than urban teaching hospitals (aOR, 0.68; all p<0.001).

Clinical comorbidities and complications were the strongest factors associated with mechanical ventilation. Each one-point increase in Elixhauser comorbidity score was associated with a 19% increase in the odds of intubation (aOR, 1.19 per point; 95% CI, 1.17-1.20; p<0.001). Sepsis (aOR, 5.42; 95% CI, 5.17-5.67), AKI (aOR, 2.35; 95% CI, 2.24-2.46), and gastrointestinal bleeding (aOR, 1.84; 95% CI, 1.75-1.94) were the most prominent complications associated with mechanical ventilation (all p<0.001). In contrast, several chronic conditions were inversely associated with intubation, including diabetes mellitus (aOR, 0.69; 95% CI, 0.66-0.73), hyperlipidemia (aOR, 0.73; 95% CI, 0.68-0.78), ascites (aOR, 0.89; 95% CI, 0.84-0.94), and AUD (aOR, 0.92; 95% CI, 0.85-0.99; all p≤0.02) (Table 4).

**Table 4:** Multivariable logistic regression of factors associated with mechanical ventilation among hospitalizations for hepatic encephalopathy. Multivariable logistic regression was used to identify factors associated with mechanical ventilation. Adjusted odds ratios (aORs) with 95% confidence intervals are reported. The model was adjusted for age, sex, race, insurance status, hospital bed size, hospital location, Elixhauser comorbidity index, alcohol use disorder, acute kidney injury, sepsis, diabetes mellitus, ascites, gastrointestinal bleeding, and hyperlipidemia. aOR: Adjusted odds ratio; AUD: Alcohol use disorder; AKI: Acute kidney injury.

Characteristic	Adjusted OR	95% CI	p-value
Age	0.99	0.98-0.99	<0.001
Sex			
Male	Reference	Reference	
Female	0.92	0.88-0.96	<0.001
Race			
White	Reference	Reference	
Black	1.17	1.08-1.26	<0.001
Hispanic	1.04	0.98-1.10	0.2
Asian or Pacific Islander	1.2	1.02-1.40	0.025
Native American	0.95	0.79-1.14	0.6
Other	1.23	1.07-1.40	0.003
Insurance			
Medicare	Reference	Reference	
Medicaid	1.15	1.08-1.23	<0.001
Private	1.13	1.06-1.21	<0.001
Other	1.23	1.13-1.35	<0.001
Hospital location/teaching status			
Urban teaching	Reference	Reference	
Rural	0.68	0.61-0.77	<0.001
Urban nonteaching	0.99	0.93-1.04	0.7
Hospital bed size			
Small	Reference	Reference	
Medium	1.14	1.07-1.23	<0.001
Large	1.18	1.10-1.26	<0.001
Elixhauser comorbidity score	1.19	1.17-1.20	<0.001
Diagnosis of AUD	0.92	0.85-0.99	0.02
AKI	2.35	2.24-2.46	<0.001
Sepsis	5.42	5.17-5.67	<0.001
Diabetes mellitus	0.69	0.66-0.73	<0.001
Ascites	0.89	0.84-0.94	<0.001
Gastrointestinal bleeding	1.84	1.75-1.94	<0.001
Hyperlipidemia	0.73	0.68-0.78	<0.001

## Discussion

Cirrhosis represents a substantial and growing global health burden, with its incidence increasing by 58.2% between 1990 and 2021, reaching an estimated 58.4 million cases worldwide and contributing to nearly 1.43 million deaths annually [10]. In the United States alone, approximately 2.2 million adults are affected, and projections suggest that this burden will continue to escalate in the absence of targeted interventions [11,4]. The high mortality associated with cirrhosis is largely attributable to its systemic complications. HE is a common and potentially fatal complication of cirrhosis. HE is defined as a reversible clinical syndrome characterized by a spectrum of cognitive, psychomotor, and psychiatric disturbances arising from acute or chronic severe liver disease [12]. Overt HE occurs in 10-14% of patients at the time of initial cirrhosis diagnosis and develops in 30-40% of patients during the course of their disease [4]. The development of HE is associated with significant impairment in quality of life, reduced prioritization for liver transplantation, and poor overall prognosis with high morbidity and mortality, independent of the MELD score [12-14]. Beyond its clinical impact, HE also imposes a considerable economic burden on the healthcare system [15]. Although most patients with HE improve with management of the underlying condition, a significant proportion require mechanical ventilation.

Our study demonstrated that the need for mechanical ventilation was associated with significantly higher odds of in-hospital mortality, a finding consistent with prior studies reporting increased mortality among patients with HE requiring intubation and ventilatory support. Gibbs JT et al. reported that 30-day survival was only 38.9% in HE patients requiring mechanical ventilation compared with 89.4% in those not requiring mechanical ventilation (p<0.0001) [16]. Fichet J et al. also demonstrated that severe HE carries a substantial mortality burden, with 35% of intensive care unit patients dying during their stay and 54% dying within one year of hospitalization; notably, 82% of these patients required mechanical ventilation [17]. Consistent with these findings, Saffo S and Garcia-Tsao G reported that 64% of patients with grade IV HE who underwent early intubation died during their hospitalization, with mechanical ventilation emerging as the strongest independent predictor of in-hospital mortality on multivariable logistic regression analysis (aOR, 3.0; 95% CI, 2.14-4.20; p<0.001) [7]. Given the poor clinical outcomes and substantial healthcare resource utilization associated with advanced HE, the need for mechanical ventilation reflects advanced disease severity and is associated with poor outcomes.

In our study, we found a nearly ten-fold increase in in-hospital mortality among patients who required mechanical ventilation compared with those who did not (43% vs. 4.7%; aOR, 8.32; 95% CI, 7.84-8.83; p<0.001), along with an approximately three-fold increase in total hospitalization costs ($48,511 vs. $15,731; adjusted RR, 2.23; 95% CI, 2.18-2.28; p<0.001). The observed differences in mortality and associated costs, when considered across a cohort exceeding 500,000 hospitalizations, reflect a substantial healthcare burden associated with critical illness requiring mechanical ventilation. These findings highlight the need to identify clinical factors associated with mechanical ventilation in patients with HE, which remain incompletely characterized in the existing literature.

Our study further investigated the most significant factors associated with mechanical ventilation in this large-scale cohort. Using multivariable logistic regression analysis, we identified sepsis (aOR, 5.42; 95% CI, 5.17-5.67; p<0.001), AKI (aOR, 2.35; 95% CI, 2.24-2.46; p<0.001), and GI bleeding (aOR, 1.84; 95% CI, 1.75-1.94; p<0.001) as the strongest factors associated with mechanical ventilation among patients hospitalized with HE. These findings are consistent with the well-established role of infections, GI bleeding, and electrolyte disturbances as recognized precipitating factors for acute episodes of overt HE. Prior studies have demonstrated that bacterial infections are significantly associated with progression to overt HE and significantly shorter time to death [18]. Early recognition and management of HE precipitating factors are important for clinical stabilization and appropriate care escalation. Our findings underscore the association of these clinical factors with mechanical ventilation in this population, as recognizing at-risk individuals may facilitate timely identification of high-risk patients and support appropriate escalation of care.

Limitations

Several limitations of this study warrant consideration. First, as with all studies utilizing administrative data, the NIS is subject to coding errors and potential misclassification of diagnoses, which may have introduced bias into the reported outcomes. ICD-10 codes may not fully capture the clinical nuances of HE severity or the precise indication for mechanical ventilation. Second, residual confounding related to liver disease severity cannot be excluded because the database lacks detailed clinical measures such as MELD score, Child-Pugh class, laboratory values, and other objective markers of hepatic function. Third, our study is unable to determine whether mechanical ventilation was initiated for airway protection in the setting of encephalopathy, respiratory failure from a separate etiology such as sepsis or aspiration pneumonia, or as part of a procedure. This limits the specificity of our findings. Although baseline comorbidity profiles were broadly comparable between groups, unmeasured differences in acute clinical severity may have influenced procedural utilization and mortality, and residual confounding cannot be excluded. Fourth, our study was limited to a predefined set of comorbidities available within the NIS dataset; a more comprehensive assessment incorporating a broader range of comorbidities may have provided additional insight and identified additional factors associated with mechanical ventilation not captured in our analysis. Although missing data were limited, the use of complete-case analysis may introduce bias if data are not missing at random. Furthermore, the observed associations are likely influenced by confounding by severity, as patients requiring mechanical ventilation represent a more critically ill subgroup with greater physiologic instability not fully captured in administrative data. In addition, the cross-sectional nature of the dataset precludes assessment of temporal relationships, raising the possibility of reverse causality between complications such as sepsis or AKI and mechanical ventilation. Moreover, the NIS does not capture goals-of-care discussions, do-not-intubate status, or palliative care involvement, which may have influenced both the rate of mechanical ventilation and observed mortality outcomes, potentially introducing bias into our analysis. Finally, the present study captures only in-hospital mortality, and post-discharge outcomes such as long-term mortality could not be assessed, which may limit the generalizability of these findings beyond the inpatient setting.

## Conclusions

Our study represents a comprehensive, large-scale nationwide cohort analysis comparing patients with HE who required mechanical ventilation with those who did not. Mechanical ventilation in patients hospitalized with HE was associated with higher in-hospital mortality, longer LOS, and increased healthcare costs. Sepsis, AKI, and GI bleeding emerged as the strongest factors associated with mechanical ventilation, underscoring the importance of early recognition and prompt management of these high-risk clinical features. These findings likely reflect the greater severity of illness in patients requiring mechanical ventilation rather than a direct causal effect. Prompt identification of at-risk patients and appropriate triage to higher levels of care, along with management of precipitating factors, may support improved risk stratification and clinical decision-making in this vulnerable population.
